# Preparation and Characterization of Ginger Essential Oil Microcapsule Composite Films

**DOI:** 10.3390/foods10102268

**Published:** 2021-09-25

**Authors:** Hua-Hua Wang, Meng-Yao Li, Zhou-Yong Dong, Tie-Hua Zhang, Qing-Yu Yu

**Affiliations:** 1 College of Food Science and Engineering, Jilin University, Changchun 130062, China; hhwang19@mails.jlu.edu.cn (H.-H.W.); li_mengyao1023@163.com (M.-Y.L.); zhangth@jlu.edu.cn (T.-H.Z.); 2 College of Biological and Agricultural Engineering, Jilin University, Changchun 130062, China; yqy@jlu.edu.cn

**Keywords:** microcapsule, edible film, ginger essential oil, gelatin, functional components

## Abstract

New food packaging has shown research significance in the face of increasing demand for high-quality foods and growing attention paid to food safety. In this study, ginger essential oil microcapsule composite films were prepared by combining microcapsules prepared by a complex coacervation method with gelatin films, and the mechanical properties and active functions of the composite films were analyzed. Fourier-transform infrared spectroscopy and differential scanning calorimetry confirmed the successful encapsulation of ginger essential oil. The scanning electron microscopy of the composite films showed the microcapsules and gelatin film matrix were highly compatible. During the entire storage period, the antioxidant capacity of the ginger essential oil microcapsule films weakened more slowly than ginger essential oil microcapsules and could be maintained at a relatively high level for a long time. The microcapsule films had excellent inhibitory effects on *Escherichia coli, Staphylococcus aureus*, and *Bacillus subtilis.* Therefore, the direct addition of microcapsules to a film matrix can broaden the application range of microcapsules and increase the duration of the release of active ingredients. Ginger essential oil microcapsule films are potential biodegradable food packaging films with long-lasting activity.

## 1. Introduction

Ginger essential oil is a volatile oily substance extracted from ginger rhizomes. Ginger contains 0.25%–0.3% volatile oil, which is mainly composed of gingerol, aromatic alcohol, and terpenoids. Experiments have proven that ginger essential oil has antioxidation, bacteriostatic, and antitumor functions [1,2,3], and it is the most important active ingredient in ginger. Avanco GB et al. studied the scavenging ability of ginger essential oil against 2,2′-azido-bis-3-ethylbenzothiazoline-6-sulfonic acid (ABTS) and 2,2-diphenyl-1-pyridylhydrazide (DPPH) free radicals, against which its half inhibitory concentration (IC_50_) was found to be 0.54 and 10.03 mg/mL, respectively [4], thus indicating that ginger essential oil has high antioxidant activity. Ginger oil has a strong inhibitory effect on *Staphylococcus aureus, Escherichia coli, Pseudomonas aeruginosa, Acinetobacter baumannii*, and 30 multi-drug resistant *A. baumannii* clinical isolates [5]. Reportedly, spraying ginger essential oil on a plant’s surface can shorten the residing time of pests and reduce the damage of pesticides to the environment and insects [6]. Ginger essential oil also has antibacterial and fresh-keeping effects on papaya that can help maintain its high quality [7]. Feeding with ginger essential oil can improve the activity of antioxidant enzymes in quails and reduce the lipid peroxidation of germ cells [8]. However, the activity of ginger essential oil can be reduced by its instability in the air, volatility, and susceptibility to temperature and humidity or oxidation, which limits its application in foods.

Microencapsulation refers to the use of one or more polymer film-forming substances (the wall material) to embed solid, liquid, or gas (the core material) to form a micro-level particle size protection termed microencapsulation [9]. Microencapsulation can protect sensitive ingredients, control the release of core materials, mask bad flavor and color, and improve the state of materials. At present, microcapsule technology is widely used in flavors and fragrances. For example, PureDelivery^®^ and Qpearl^®^ of Givaudan (the world’s largest flavor and fragrance company) and Firmenich (a fragrance company) have series of slow-release flavor microcapsule products. Fernandes et al. studied the effects of different proportions of gum Arabic, maltodextrin, and inulin as wall materials on the preparation of ginger essential oil microcapsules [10]. The microspheres produced by the gum Arabic and maltodextrin mixture had higher wettability, encapsulation efficiency, and absorption than the others. Zhang et al. used ginger essential oil/beta cyclodextrin/chitosan to prepare spheres and characterized them by inclusion, ionic gel, and spray drying methods [11]. Racoti et al. prepared and characterized PMMA and ginger essential oil microcapsules by suspension polymerization [12]. Simon Brown et al. developed microcapsules of ginger extract by maltodextrin and/or gum Arabic and determined its preparation method [13]. It was found that ginger essential oil microcapsules can be added as a raw material to foods to prevent oxidation and deterioration. Riarita et al. studied the ability of the encapsulation of ginger essential oil in different proportions of gum Arabic to improve the solubility and antibacterial activity of ginger microcapsules, and they compared the antibacterial activities of different gum Arabic microcapsules against *E. coli* and *S. aureus* [14].

Nowadays, most food packaging materials are synthetic plastic films prepared from fossil fuels. However, synthetic plastic packaging films have many disadvantages, such as non-degradability (which causes considerable pressure and burden on the natural environment) and the possession of bisphenol A, phthalate, and other chemicals that have hidden dangers to human health during use. Therefore, the development of green, safe, and degradable edible food packaging films has attracted researchers. Edible films are formed by the combination, heating, pressing, coating, and extrusion of edible materials that are mainly composed of natural macromolecular substances (e.g., lipids, proteins, and polysaccharides) and other natural macromolecules or biopolymers with the addition of edible plasticizers and crosslinkers. Edible films can separate air and water on the surface of or inside foods in the form of spraying, dipping, and wrapping in order to protect them and prolong their shelf lives. Clove essential oil and cinnamon essential oil have high antibacterial activities and are widely used in antibacterial films [15]. Many studies have demonstrated that ginger essential oil can enhance the antioxidant capacities of chitosan carboxymethylcellulose sodium/montmorillonite composite films and gelatin-based films [16,17,18]. Biodegradable films containing active substances such as plant essential oils have more effective antioxidant and antibacterial properties. However, the volatility, instability, strong smell, and oxidizability of their active substances limit the practical application of biodegradable films in the food industry. The use of microcapsule technology to embed active substances and prepare edible films can release active ingredients for long durations [19,20]. For instance, Yuan et al. prepared composite films of thymus oil microcapsules to study the slow release, antibacterial properties, and applications of the composite films on chilled meat; they achieved promising results [21]. De Medeiros et al. studied the addition of oregano essential oil microcapsules to starch films and characterized the microcapsules and composite films. Their results showed that the films containing microencapsulated oregano essential oil had inhibitory activity against *Staphylococcus aureus* [22].

Ginger essential oil has excellent antioxidant and antibacterial properties. Theoretically, microencapsulation can moderately delay the release of essential oil. Adding microcapsules containing essential oil into a film matrix can not only improve the sustained-release effect of active substances and the long-term effectiveness but also broaden the use of essential oil microcapsules. However, there is no research on ginger essential oil microcapsule films. Therefore, in this study, ginger essential oil–gelatin–gum Arabic microcapsules were prepared and added to gelatin films to prepare composite films. The performance of the films was verified and indicated that green edible films suitable for long-term food preservation can be prepared.

## 2. Materials and Methods

### 2.1. Materials

Ginger essential oil (≥99%) was obtained from Kangmin Materia Medica Refinery in Jiangxi Province. Gelatin, gum Arabic, glacial acetic acid, sodium hydroxide, and glycerin (all analytically pure) were purchased from Beijing Chemical Plant. Glutamine transaminase (food grade) was bought from Nanning Pangbo Biological Engineering Co., Ltd. Nanning, China.

### 2.2. Preparation of Ginger Essential Oil Microcapsules

According to the method of da Cruz [23] with some modifications, microcapsules were prepared by a complex coacervation method with gelatin–gum Arabic as the wall material and ginger essential oil as the core material. The specific preparation method determined on the basis of preliminary experimental results is as follows. First, a gelatin–gum Arabic 1:1 (*w/w*) solution with total wall material concentration of 1.0% was prepared, heated, and stirred for full dissolution. Then, ginger essential oil was added at a core-to-wall ratio of 2:3 and homogenized for 3 min with a high-speed homogenizer at 10,000 r/min to form a homogeneous emulsion. This emulsion was stirred at 45 °C at a rate of 400 r/min and then adjusted to pH 4 by slowly adding a 1 mol/L HCl solution. Microcapsules were initially formed when the system became visibly turbid under the naked eye. Then, stirring was continued for 30 min to ensure complete reaction. After that, the suspension was further stirred, cooled in an ice water bath until reaching 15 °C, and adjusted to pH 6.0 by adding a 1 mol/L NaOH solution. Glutamine aminotransferase (0.3 g/g gelatin) was added to the suspension, which was solidified in the ice water bath for 3 h and equilibrated at 4 °C for 24 h. Then, the microcapsule suspension was filtered, and residues were collected in the form of wet microcapsules of ginger essential oil; these were oven-dried at 45 °C for 6 h to form dry powder (oil loading rate at 45.29% (*w/w*)), followed by refrigeration at 4 °C for further use.

### 2.3. Preparation of Ginger Essential Oil Microcapsule Composite Films

#### 2.3.1. Preparation of Microcapsule Films

A ginger essential oil microcapsule suspension concentrate was prepared in advance. To ensure that the microcapsules more evenly distributed in the film matrix following the method in Section 2.2, the liquid phase in the microcapsule suspension was filtered out using a Brinell funnel and a vacuum pump, and the volume was concentrated to the original 1/6. In this way, the influence of the residual wall material in the suspension on the microcapsule films was reduced.

Ginger essential oil microcapsule films were prepared according to the method of EOM H [24] with appropriate modifications. Specifically, an amount of gelatin was weighed, added to a proportion of glycerol, and put into a beaker containing 100 mL of deionized water for 2 h. The beaker was placed in a constant-temperature magnetic stirrer, and the mixture was dissolved under stirring at 50 °C for 15 min to form a gelatin solution. Then, a proportion of microcapsules was added and heating was stopped, with stirring for another 2 min. The beaker was put in a vacuum-drying oven for degassing until no obvious bubbles floated up. The film solution (50 mL) was poured into an 8 × 15 cm^2^ plexiglass formwork, shaken gently to make the film liquid uniform, and allowed to stand at 4 °C for 30 min. The film liquid transitioned to a gel state and dried at 20 °C for 48 h. Before determination, the films were placed in an environment with a constant temperature of 25 ± 1 °C and humidity of 50 ± 5% for 48 h to reach equilibrium.

#### 2.3.2. Optimization of Preparation Conditions of Ginger Essential Oil Microcapsules Films

Composite films of ginger essential oil microcapsules were prepared according to the process described in Section 2.3.1. With the elongation at break and thickness as indexes and other test conditions remaining stable, we changed and studied gelatin addition (2, 4, 6, 8, and 10 g/100 mL), gelatin:glycerin ratio (1:0.2, 1:0.3, 1:0.4, and 1:0.5) (*w/w*), microcapsule addition (0, 0.34, 0.85, 1.36, 1.87, and 2.38 g/100 mL) (*w/w*), and drying condition (20, 30, and 40 °C).

### 2.4. Microcapsule Characterization

#### 2.4.1. Fourier-Transform Infrared Spectroscopy (FTIR)

Based on the test method of Zhang et al. [25], FTIR was used to analyze the gelatin, gum Arabic, mixture of gelatin and gum Arabic, ginger essential oil, and microcapsules. The scanning wavenumber range was set to 400–4000 cm^−1^ at a resolution of 4 cm^−1^, and scanning was carried out 32 times. In brief, an amount of a sample was weighed, mixed with KBr, and fully ground (because the strong water absorption ability of KBr would have affected the experimental results, the experimental process was kept as short as possible). Then, the tablets were pressed with a tablet press and put into an infrared spectrometer for scanning.

#### 2.4.2. Differential Scanning Calorimetry (DSC)

According to the method of Arfat et al. [26], the thermal properties of ginger essential oil, gelatin, gum Arabic and microcapsules were analyzed by DSC in a nitrogen atmosphere. Briefly, a sample (6.20 mg) was accurately weighed, put in a crucible, pressed, and scanned at the heating rate of 10 °C/min from 30 to 400 °C. A crucible without any sample was set as a blank control.

### 2.5. Characterization of Composite Films of Ginger Essential Oil Microcapsules

Microcapsule films intended for use as food packaging materials should have high antioxidative and antibacterial physiological activities, be more widely used in foods, and meet good mechanical performance requirements. Therefore, a corresponding performance test was conducted in this experiment. For the optimized experimental conditions, the dosages of gelatin and microcapsules were 6 and 1.36 g/100 mL of film liquid, respectively; the ratio of gelatin:glycerol was 1:0.3; and the drying process occurred at 20 °C for 48 h. Under these conditions, the microcapsule films were observed by scanning electron microscopy (SEM), and the antioxidant and antibacterial properties were analyzed.

Before the performance test and characterization, the microcapsule films were placed in a constant temperature (25 ± 1 °C) and humidity (50 ± 5%) environment for 48 h to reach equilibrium.

#### 2.5.1. Thickness Measurement

Film thickness was measured with a screw micrometer. The center and five different points around each microcapsule film were measured, and the average value of the results was taken.

#### 2.5.2. Determination of Mechanical Properties

The mechanical properties of the films were detected by referring to the test method of Anker et al. [27] with slight adjustments. In brief, a microcapsule film was cut into samples of 150 × 20 mm^2^. The edges of the sample were complete and smooth without gaps to reduce the cutting-caused deviation of test data. At a speed of 60 mm/min and a clamping distance of 50 mm, 4 samples of each microcapsule film were taken for longitudinal and transverse tensile strength measurements, and the results were averaged.

Tensile strength *TS* (MPa) was calculated as follows:

(1) $$TS=\frac{F}{b\times d}$$

*F*—maximum tension at break (N);

*b*—width of sample (mm);

*d*—thickness of sample (mm).

Elongation at break *E* (%) was calculated as follows:

(2) $$E\left(\%\right)=\frac{\Delta L}{L}\times 100$$

Δ*L*—elongation at break (mm);

*L*—original length (mm).

#### 2.5.3. Oxygen Permeability Test

Based on the method of Wang et al. [28] with modifications, the oxygen permeability of the films was tested with a GDP-C permeability tester. Under constant temperature (25 ± 1 °C) and constant humidity (50 ± 5%), a film was placed in the sample chamber with an exposure area of 100 cm^2^. Under these conditions, 99% pure oxygen was injected at the top and bottom.

### 2.6. SEM

Based on the method of Cai et al. [29] with slight changes, the morphology of microcapsules and the surface and cross-section of microcapsule films were observed with SEM. The acceleration voltage was set at 5.0 kV, and the observation time was kept as short as possible in order to reduce the damage of the blurred field of view caused by the long-term irradiation of the electron beams.

Microcapsule sample preparation: a layer of conductive glue was put on the copper sheet, and a drop of microcapsule suspension was taken on the surface for air drying. Then, gold was sprayed on the sample for observation.

Film sample preparation: each newly-prepared microcapsule film was cut into a 5 × 5 mm^2^ sample, which was spread evenly on a slide stuck with conductive adhesive. After gold spraying, the surface structure of the film was observed.

### 2.7. Determination of Antioxidant Properties

Ginger essential oil solution sample preparation: an amount of ginger essential oil was accurately weighed and put into 10% ethanol. Then, a ginger essential oil solution with a mass concentration of 1 mg/mL was configured.

Microcapsule sample preparation: an amount of microcapsule powder was accurately weighed (calculated as per the mass of the core material; the microcapsule oil load rate was calculated as 45.29%), added to 10% ethanol, and configured to contain the same mass of ginger essential oil (1 mg/mL) in a stock solution.

Antioxidation sample preparation of microcapsule films: with 10% ethanol as the solvent, the microcapsule films were cut into squares containing the same quantity of ginger essential oil (1 mg/mL) and placed in the solvent.

A sample solution (2 mL) was taken from each of the abovementioned samples at a time interval, shaken gently, and centrifuged at 8000 r/min for 1 min to form a clear sample solution. The DPPH radical (DPPH^·^) and ABTS radical (ABTS^+^) scavenging rates were measured.

(1) Determination of DPPH^·^ scavenging rate

We used Esmaeil’s [30] method with slight modifications to determine the DPPH^·^ scavenging rate. First, 2 mL of a 0.1 mol/L DPPH–ethanol solution (DPPH dissolved in 95% ethanol solution) were added to the sample supernatant after centrifugation (8000 r/min for 5 min), shaken, mixed with force, and allowed to react in the dark for 30 min. The absorbance of the mixed solution at 517 nm was measured with an ultraviolet–visible spectrophotometer. A DPPH blank (2 mL of DPPH–ethanol and 2 mL of 10% ethanol) was also tested. The DPPH^·^ scavenging rate was calculated as follows:

(3) $$\mathrm{DPPH}\;\mathrm{radical}\;\mathrm{scavenging}\;\mathrm{rate}\;\left(\%\right)=\frac{A_{0}-A}{A_{0}}\times 100$$

where: $A_{0}$ is the absorbance of 2 mL of DPPH–ethanol and 2 mL of 10% ethanol;

A—absorbance of 2 mL of ethanol and 2 mL of sample solution.

(2) Determination of ABTS^+^ scavenging rate

P.A. Loyeau’s [31] method with slight modifications was referred to for the determination of the ABTS^+^ scavenging rate. An ABTS^+^ stock solution was prepared by mixing 5 mL of a 7 mmol/L ABTS^+^ solution with 88 μL of a 140 mmol/L K_2_S_2_O_8_ solution, placing it in the dark at room temperature overnight, and equilibrating it. Before measurement, 95% ethanol was used for dilution to ensure an absorbance of 0.70 ± 0.02 at 734 nm.

During the measurement, 2 mL of the ABTS^+^ stock solution was added to the tested sample after centrifugation, and the absorbance was measured by the UV–Vis spectrophotometer after 6 min, which was recorded as *A*_1_. After centrifugation, the absorbance of sample and 2 mL 95% ethanol was recorded as *A*_2_, and the absorbance of 2 mL ABTS^+^ stock solution and 2 mL 10% ethanol solution was recorded as *A*_3_. The ABTS^+^ scavenging rate was calculated as:

(4) $$\mathrm{ABTS}\;\mathrm{radical}\;\mathrm{scavenging}\;\mathrm{rate}\left(\%\right)=(1-\frac{A_{1}-A_{2}}{A_{3}})\times 100$$

where: $A_{1}$—absorption of 2 mL sample solution and 2 mL ABTS^·+^ stock solution;

$A_{2}$—absorbance of 2 mL sample solution and 2 mL 95% ethanol;

$A_{3}$—absorbance of 2 mL ABTS^+^ stock solution and 2 mL 10% ethanol.

### 2.8. Determination of Antibacterial Activity

All the inoculation and dilution processes were carried out on a sterile operating platform with 40 min before UV sterilization. The *E*. *coli*, *S*. *aureus*, and *B*. *subtilis* stored in the inclined plate were inoculated on a solid medium and separated by lines. They were cultured upside down in a biochemical incubator at 37 °C for 24 h. In an aseptic environment, single colonies with good morphology on each solid medium were scraped with an inoculation ring and placed in 20 mL of liquid medium for 12 h in a constant-temperature shaking incubator (37 °C at 200 r/min). A bacterial suspension with concentration of 10^6^–10^7^ CFU/mL was prepared for use and compared with Macintosh turbidimetric tubes.

The abovementioned bacteria suspension (150 μL) was evenly coated onto a plate. Sterilized transparent glass tubes (outer diameter: 5.50 ± 0.02 mm) were used for drilling, and the medium at the drilling place was picked up with inoculation needles. Each plate was evenly drilled with 4 holes; the distance between holes was not less than 30 mm, and the distance between the hole center and the medium edge was not less than 10 mm. The ginger essential oil microcapsule powder (sterilized under an ultraviolet lamp for 40 min in advance, with the sterile glass rod turned over several times) was accurately weighed (5, 10, and 15 mg) and gently transferred into wells. Then, 10 mg of an empty capsule were added into the remaining well. After the addition of microcapsules, one drop of an agar medium at about 45 °C was added into each well for sealing to prevent the microcapsules from falling off. After 24 h of inverted culture at 37 °C in a biochemical incubator, the diameter of each inhibition zone was measured with a vernier caliper.

Under aseptic conditions, 0.2 mL of the abovementioned bacteria suspension was aspirated by pipettes, evenly spread on a medium plate, and allowed to stand for 10 min. The microcapsule films were cut into 4 × 4 cm^2^ squares, which were irradiated on both sides for 30 min under a UV sterilization lamp in advance. Then, the microcapsule films were put into the plate containing bacteria with sterile tweezers, and a piece of a microcapsule film was placed in the center of each culture dish. The dish was covered, inverted, and cultured in a biochemical incubator at 37 °C for 24 h. The effects of the front and bottom sides of the films on microorganism growth in the medium were observed to evaluate the antibacterial effect of the microcapsule films.

### 2.9. Statistical Analysis

Data analysis was carried out with Origin (ver. 6.0, IBM software, Chicago, IL, USA). Each experiment was repeated three times. A Duncan test and one-way analysis of variance were used. Significance was defined as *p* < 0.05.

## 3. Results and Discussion

### 3.1. Characterization of Microcapsules

#### 3.1.1. FTIR

**Figure 1:**
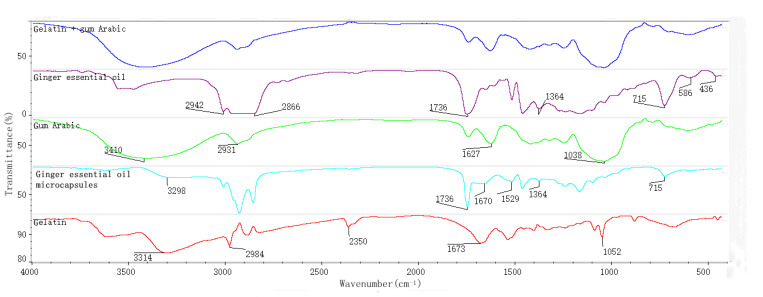
FTIR spectra of gelatin, gum Arabic, gelatin and gum Arabic, ginger essential oil, and giner essential oil microcapsules.

Gelatin is an amphoteric polymer composed of polypeptides with different molecular weights (Figure 1). The FTIR spectrum of gelatin showed a strong characteristic peak of amino near 3314 cm^−1^, a C–H expanding vibration peak of alkane at 2984 cm^−1^, an expanding vibration peak of amido carbonyl at 1673 cm^−1^, and a C–H bending vibration at 1052 cm^−1^ [32,33].

Gum Arabic is a complex mixture of polysaccharides and glycoprotein. The FTIR spectrum of gum Arabic showed a strong hydroxyl characteristic peak at about 3410 cm^−1^, a C–H stretching vibration peak of CH_2_ or CH_3_ at about 2931 cm^−1^, a COO^−^ stretching vibration peak at 1627 cm^−1^, and a CO bending vibration peak near 1038 cm^−1^. The characteristic absorption band of the amino group in gum Arabic at 3400–3500 cm^−1^ must have been covered by the broad band of OH [34,35].

The C=C stretching peaks of ginger essential oil at 1736 and 1364 cm^−1^ were related to C–H bending, and the peaks at 2942 and 2866 cm^−1^ showed the vibration modes of CH_2_ and C–C–H, respectively.

The spectrogram of the physical mixture of gelatin and gum Arabic was very similar to that of gum Arabic. In addition, the peak at 2350 cm^−1^ in gelatin disappeared here. Compared with the physical mixture, new peaks appeared at 3298, 1670, and 1529 cm^−1^ in the spectrum of the microcapsules, and the original peaks of essential oil at 586 and 436 cm^−1^ disappeared. One reason for this is that the microcapsules contained ginger essential oil, and a new chemical bond was formed between the wall and core materials. In addition, compared with ginger essential oil, the peaks of the microcapsules at 1736, 1364, and 715 cm^−1^ were significantly reduced in intensity, indicating that the wall and core materials in the microcapsules did not simply physically mix. These results confirm the successful encapsulation of ginger essential oil.

#### 3.1.2. DSC

**Figure 2:**
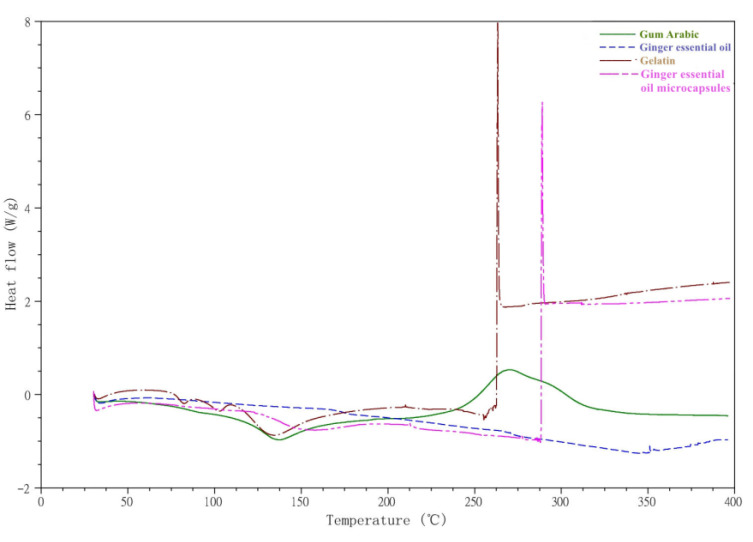
DSC curves of gelatin, gum Arabic, ginger essential oil, and ginger essential oil microcapsules.

Endothermic peaks of gelatin appeared at 75–100, 100–110, and 125–150 °C (Figure 2), which were caused by water loss. The large exothermic peak between 250 and 275 °C was due to the heat of gelatin cracking [36].

The DSC curve of gum Arabic showed an endothermic peak at 125–150 °C, and the exothermic peak at 250–275 °C may have been related to the melting and partial thermal decomposition of polysaccharides [37].

The exothermic peak of ginger essential oil near 350 °C was caused by the loss of essential oil quality.

An endothermic peak at about 150 °C in the microcapsules was related to gum Arabic and gelatin. The enthalpy peak of microcapsules at about 150 °C was lower than that of the wall material, indicating that the stability of microcapsules was higher. In addition, a new exothermic peak appeared between 275 and 300 °C in the microcapsules, but (in contrast to ginger essential oil) there was no peak near 350 °C. This indicates that the encapsulation of ginger essential oil in microcapsules is not simple but some micro changes occurred, which is consistent with the infrared spectra.

### 3.2. Optimization of Preparation Conditions of Ginger Essential Oil Microcapsules Films

(1) Added amount of gelatin

When the dosage of gelatin was 2 or 10 g/100 mL, no complete and uniform microcapsule film could be obtained (Table 1). When the dosage of gelatin was too small, the microcapsule films were too thin and did not easily fall from the mold. When the dosage of gelatin was too large, however, the film liquid was too viscous to completely degas. Due to the influence of air bubbles, the thicknesses of each part of the dried microcapsule film and the distribution of microcapsules were uneven. As the dosage of gelatin increased within 4–8 g/100 mL, a more complete and uniform microcapsule film could be obtained and the films became thicker. When the dosage was 6 g/100 mL, the elongation at break was the largest (45 ± 0.5%). Therefore, the optimal amount of gelatin in this formulation is 6 g/100 mL.

(2) Gelatin:glycerin ratio

When the gelatin:glycerol ratio was 1:0.5, no ideal microcapsule films could be obtained (Table 1) because of the severe deformation occurring during demolding. When the gelatin:glycerin ratio was 1:0.2–1:0.4, a relatively uniform and complete microcapsule film could be obtained, and when the ratio gradually rose, the breaking elongation of the microcapsule films increased. As previously reported, the dosage of glycerin is linearly related to the elongation of protein films in a range [38], but in our study, the increase in elongation at break was not regular, potentially because the presence of capsule microparticles moderately influenced the crosslinking between gelatin molecules. The results were similar when the gelatin:glycerin ratio was 1:0.3 and 1:0.4, but glycerin has a hygroscopicity that increases the hygroscopicity of microcapsule films and is thus inconducive to storage. Therefore, the optimal gelatin:glycerin ratio in this formulation is 1:0.3.

(3) Drying conditions

As described in the preparation method section, after degassing and inversion, the film liquid was allowed to stand at 4 °C for 30 min to allow it to transition to the gel state; then, drying was performed. Under drying temperatures of 30 and 40 °C and drying times of 12 and 30 h, we were unable to obtain a uniform microcapsule film. When drying for 20 °C at 48 h, the film liquid was in a gel state, the microcapsules were relatively dispersed, and the microcapsule films were relatively uniform. Therefore, the optimal drying condition for this formulation is 20 °C for 48 h.

(4) The dosage of microcapsules

The content of microcapsules directly affects the functional properties of gelatin films and was thus an important factor to be studied here. When the dosage of microcapsules was 2.38 g/100 mL, no ideal microcapsule films could be prepared (Table 1). A microcapsule connection area appeared on the surface of the dried films, and this part was texturally brittle and could not be completely removed from the mold. When the dosage was 0.34, 0.85, 1.36, or 1.87 g/100 mL, a relatively uniform and complete microcapsule film could be obtained. Therefore, these four dosages were used to further study and analyze the mechanical properties and the oxygen permeability of the microcapsule films.

### 3.3. Mechanical Properties and Oxygen Permeability of Ginger Essential Oil Microcapsule Composite Films

The elongation at break reflects the ductility and brittleness of films. When the dosage of ginger essential oil microcapsules rose, the elongation at break of the microcapsule films gradually decreased. When the amount of microcapsules was 1.87 g/100 mL, the elongation at break of the microcapsule films was significantly weakened (Table 1). This phenomenon may have been caused by the fact that when the dosage of ginger essential oil microcapsules increased, the microcapsules were less well-combined with the gelatin film matrix, resulting in significant decreases in mechanical properties. The effect of microcapsules on film thickness also validated this reasoning: when the dosage was large, the microcapsules could not be well-combined with the gelatin film matrix, so the thickness of the microcapsules rapidly increased.

Tensile strength mainly reflects the strength of microcapsule films. As the amount of ginger essential oil microcapsules increased, the tensile strength of the microcapsule films gradually decreased. When the dosage was 1.87 g/100 mL, the tensile strength of the microcapsule films obviously declined (Table 2).

The oxygen permeability of the microcapsule films first decreased and then increased as the dosage of ginger essential oil microcapsules rose (Table 2). When the dosage of microcapsules rose from 0 to 0.85 g/100 mL, the oxygen permeability gradually dropped from 2.3 to 1.5 (10^−5^ cm^3^ m^−2^ d^−1^ Pa^−1^), but when the dosage increased to 1.36 g/100 mL, no significant difference from the previous level of oxygen permeability was found. When the dosage rose to 1.87 g/100 mL, the oxygen permeability was significantly raised to 3.1 (10^−5^ cm^3^ m^−2^ d^−1^ Pa^−1^). This phenomenon may have been caused by the fact that as the amount of microcapsules increased, the microcapsule films became thicker—thus extending the diffusion path of gas molecules in the films. In comparison, the microcapsules at small dosages could be well-combined with the film matrix, thus forming denser microcapsule films. Both of the above mentioned reasons hindered the movement of gas molecules in the microcapsule films. Thus, when the dosage of the microcapsules was within a specified range, the oxygen permeability of the microcapsule films decreased with the increase of the dosage of the microcapsules. Excessive addition may have caused the ginger essential oil microcapsules to agglomerate in the gelatin film matrix, resulting in inadequate bonding on the two-phase interface and the formation of some defects such as cracks. Consequently, the gas molecules in the gelatin films easily diffused in the matrix, leading to a significant increase in oxygen permeability.

### 3.4. SEM

**Figure 3:**
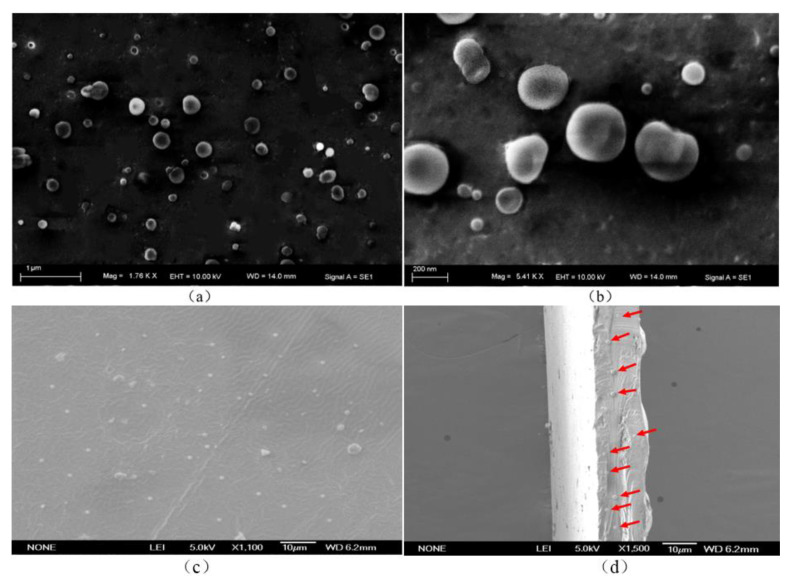
SEM images of (**a**) microcapsules (magnification: 1760×), (**b**) microcapsules (magnification: 5410×), (**c**) ginger essential oil microcapsule films (surface morphology), and (**d**) ginger essential oil microcapsule films (section morphology).

Figure 3a is an SEM image of ginger essential oil microcapsules magnified by 1760 times. The distribution of microcapsules was relatively uniform without obvious agglomeration, and the sizes were relatively consistent. Figure 3b is an SEM image obtained by magnifying 5410 times. The microcapsules can be seen to have had an approximate particle size of 300 nm and a smooth surface, as well as to have been unbroken with occasional surface wrinkles. The gelatin–gum Arabic combination was relatively cross-linked, which could protect the ginger essential oil. The microstructure and surface morphology of microcapsules are greatly affected by different preparation processes and drying methods [39]. Studies have proven that the temperature, agitation rate, and wall material concentration of a system when coagulation reaction occurs all moderately affect the particle size and morphology of microcapsules [40,41,42]. Most microcapsules produced by the spray drying process have wrinkles and depressions, because the rapid dehydration of microcapsules during spray drying induces changes in the morphology of capsule walls [43,44].

The microtopography and surface morphology of the composite films are shown in Figure 3, which shows that the microcapsules were evenly distributed on the surface of the composite films without agglomeration (Figure 3c), indicating that the amount of ginger essential oil microcapsules was appropriate. The cross-sectional view (Figure 3d) also shows that the microcapsules were uniformly distributed at the cross-section, suggesting the microcapsules and the gelatin film matrix are compatible under this preparation process and can be evenly distributed inside the films. This also verifies that microcapsules affect the mechanical properties of films because of the distribution of microcapsules inside the films.

### 3.5. Analysis of Antioxidant Properties

**Figure 4:**
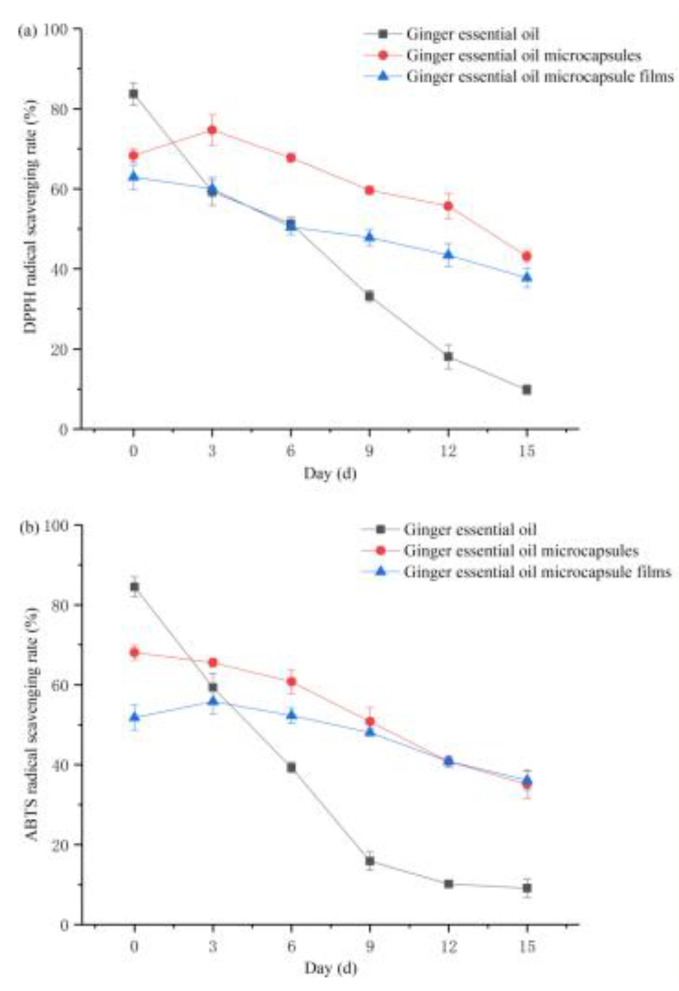
Analysis of antioxidant properties: (**a**) DPPH radical scavenging rate; (**b**) ABTS radical scavenging rate.

DPPH^·^ is often used to detect the antioxidant capacity of active substances, and it can be used to speculate the scavenging rate of aromatic radicals [45]. The ABTS^+^ detection method is also commonly used, and the two methods can be combined to comprehensively evaluate antioxidant resistance and oxidizing ability. Figure 4 shows the changes of the antioxidant capacity of ginger essential oil, ginger essential oil microcapsules, and ginger essential oil microcapsule films within 15 days. Regarding the DPPH^·^ and ABTS^+^ scavenging rates, ginger essential oil had the highest activity on the 0th day, followed by ginger essential oil microcapsules and finally ginger essential oil microcapsule films. During the entire storage period, the antioxidant capacity of the ginger essential oil microcapsule films weakened the most slowly, while that of ginger essential oil weakened the most quickly. On the 15th day of storage, the ABTS^+^ scavenging rate of the ginger essential oil microcapsule films was higher than that of the microcapsules.

This was mainly because the level of antioxidant capacity depended on the amount of active substances in ginger essential oil released into the medium in the system. Larger amounts of ginger essential oil in the medium led to better antioxidant effects. The ginger essential oil contained a high content of essential oils in the early stage, so its antioxidant capacity was high. However, because the ginger essential oil was unstable, the oil droplets floated up with the air and caused the oxidation of essential oils or the essential oil evaporated into the air and caused some loss, so the antioxidant capacity of the ginger essential oil was rapidly weakened. In the ginger essential oil microcapsule films, the ginger essential oil penetrated the wall material to reach the film matrix, further penetrated into the medium, and was then released completely. In this way, the loss induced by the “burst release” (which was caused by the high concentration of essential oils in the initial stage) was reduced, and the release of essential oils was made smoother and more sustainable. Thus, the antioxidant capacity of the ginger essential oil microcapsule films can be maintained at a relatively high level for a long time.

### 3.6. Analysis of Antibacterial Activity

Ginger essential oil microcapsules were found to have inhibitory effects on the growth and reproduction of the three tested bacteria (Table 3). The diameter of the inhibition zone was 5.72–8.14 mm, and when more microcapsules were added, the inhibitory effect was more obvious (*p* < 0.05). Among the three tested bacteria, the inhibitory effect of ginger essential oil microcapsules was the best on *E*. *coli* and the worst on *S*. *aureus*. Similarly, another study demonstrated that the inhibitory ability of ginger essential oil on Gram-negative bacteria is greater than that on Gram-positive bacteria [46]. Gram-negative bacteria have thin cell walls and a thin and loosely-crosslinked peptidoglycan layer. The active components in essential oil easily destroy the structure and inhibit the growth and reproduction of Gram-negative bacteria.

**Figure 5:**
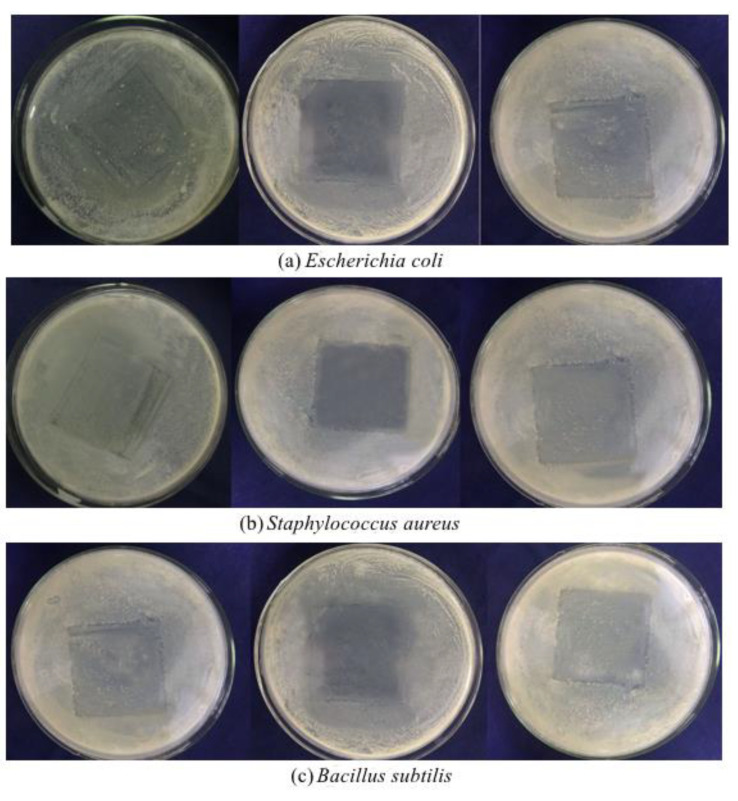
Results of the measurement of the antimicrobial ability of the microcapsule films: (**a**) *Escherichia coli*; (**b**) *Staphylococcus aureus*; (**c**) *Bacillus subtilis*. (the contact surfaces of the microcapsule films and the culture medium from left to right are: blank films, the front surface of the microcapsule films, and the bottom surface of the microcapsule films).

The inhibition capacity of the ginger essential oil microcapsule composite films against *E*. *coli*, *S*. *aureus*, and *B*. *subtilis* is shown in Figure 5, where the left picture is a control group with a blank gelatin film. Clearly, the white colonies in the square area covered by the gelatin films were slightly reduced, possibly because the gelatin films form a barrier against the growth and reproduction of microorganisms. The ginger essential oil microcapsule films had obvious inhibitory effects on *E*. *coli*, *S*. *aureus,* and *B*. *subtilis*. The antibacterial effect of the front side of the microcapsule films was significantly better than that at the bottom side because the microcapsules were more distributed on the single side surface (front side) of the films. At the same time, the core material was released from the inside of the film to both sides, so the other side (bottom) was endowed with a poor bacteriostatic ability.

## 4. Conclusions

Ginger essential oil microcapsules were prepared with microcapsule technology. The successful encapsulation of ginger essential oil was confirmed via oil loading rate determination, Fourier-transform infrared spectroscopy, and differential scanning calorimetry. The wall material and ginger essential oil in the microcapsules were not physically mixed, but microstructural changes still occurred. The preparation process of ginger essential oil microcapsule composite films was optimized. When the final gelatin concentration was 6 g/100 mL, the dosage of microcapsules was 1.36 g/100 mL, the gelatin and glycerol ratio was 1:0.3, and the drying condition was 20 °C for 48 h, ideal microcapsule films were obtained. Then, the effects of microcapsule contents on the mechanical properties and oxygen permeability of the microcapsule films were determined. The elongation at break and tensile strength of the microcapsule films gradually decreased with the increase of the dosage of the ginger essential oil microcapsules. De Medeiros et al. showed that the presence of oregano essential oil microcapsules reduced the elongation at break and tensile strength of films [22]. Their results were similar to those of our study. The oxygen permeability decreased with the increase in the dosage of ginger essential oil microcapsules, though it increased sharply when the dosage was too large. SEM showed that the microcapsules were evenly distributed on the surface of the composite films, and the gelatin film matrix was compatible with the ginger essential oil microcapsules. The microcapsule films had antioxidant capacity that changed little with time and was maintained at a high level for a long duration. Similar to the results of this study, Yuan et al. showed that the microcapsule films of thymus oil have a good sustained release effect [21]. Bacteriostatic tests showed that the microcapsule films had an inhibitory effect on *E*. *coli*, *S*. *aureus,* and *B*. *subtilis*, and the antibacterial ability of front microcapsule films was found to be significantly greater than that of the bottom side. However, compared to the microcapsule films, the gelatin films only had a slight barrier effect and could not significantly inhibit the growth of microorganisms.

## Figures and Tables

**Table 1:** Optimization of preparation conditions of ginger essential oil microcapsules films.

Gelatin (g/100 mL)	Gelatin: Glycerin Ratio	Drying Conditions		Ginger Essential Oil Microcapsules (g/100 mL)	Elongation at Break (%)	Thickness (mm)
		Temperature (℃)	Time (h)			
2	1:0.3	20	48	1.36	——	——
4	1:0.3	20	48	1.36	40.5 ± 0.9 ^b^	0.14 ± 0.03 ^c^
6	1:0.3	20	48	1.36	45.0 ± 0.5 ^a^	0.21 ± 0.02 ^b^
8	1:0.3	20	48	1.36	40.0 ± 0.8 ^b^	0.29 ± 0.02 ^a^
10	1:0.3	20	48	1.36	——	——
6	1:0.2	20	48	1.36	19.2 ± 0.5 ^c^	0.20 ± 0.02 ^c^
6	1:0.3	20	48	1.36	45.0 ± 0.5 ^b^	0.21 ± 0.02 ^b^
6	1:0.4	20	48	1.36	47.2 ± 0.6 ^a^	0.22 ± 0.01 ^a^
6	1:0.5	20	48	1.36	——	——
6	1:0.3	20	48	1.36	45.0 ± 0.5	0.21 ± 0.02
6	1:0.3	30	30	1.36	——	——
6	1:0.3	40	12	1.36	——	——
6	1:0.3	20	48	0.34	59.3 ± 1.0 ^a^	0.13 ± 0.02 ^d^
6	1:0.3	20	48	0.85	47.9 ± 0.8 ^b^	0.18 ± 0.02 ^c^
6	1:0.3	20	48	1.36	45.0 ± 0.5 ^c^	0.21 ± 0.02 ^b^
6	1:0.3	20	48	1.87	20.4 ± 1.4 ^d^	0.29 ± 0.02 ^a^
6	1:0.3	20	48	2.38	——	——

Note: “——”means that complete and uniform microcapsule films could not be obtained. Different letters indicate significant differences in data between different levels in the same column (*p* < 0.05).

**Table 2:** Tensile strength and oxygen permeability of ginger essential oil microcapsule films.

Ginger Essential Oil Microcapsules (g/100 mL)	Tensile Strength (MPa)	Oxygen Permeability (10^−5^ cm^3^ m^−2^ d^−1^ Pa^−1^)
0	35.45 ± 0.25 ^a^	2.3 ± 0.1 ^b^
0.34	30.04 ± 0.41 ^b^	2.0 ± 0.1 ^c^
0.85	26.51 ± 0.34 ^c^	1.5 ± 0.2 ^d^
1.36	25.03 ± 0.65 ^d^	1.4 ± 0.1 ^d^
1.87	19.19 ± 0.50 ^e^	3.1 ± 0.2 ^a^

Note: Different letters indicate significant differences in data between different levels in the same column (*p* < 0.05).

**Table 3:** Antibacterial test of ginger essential oil microcapsules.

**Dosage of Microcapsules** **(mg)**	**Diameter of Bacteriostatic Zone (mm)**		
	*Escherichia coli*	*Staphylococcus aureus*	*Bacillus subtilis*
0	——	——	——
5	6.19 ± 0.38 ^a^	5.72 ± 0.21 ^a^	5.99 ± 0.23 ^a^
10	6.94 ± 0.28 ^b^	6.15 ± 0.19 ^b^	6.82 ± 0.26 ^b^
15	8.14 ± 0.33 ^c^	7.28 ± 0.30 ^c^	7.69 ± 0.35 ^c^

Note: “——” means no bacteriostatic zone was detected. Different letters indicate significant differences in data between different levels in the same column (*p* < 0.05).

## Data Availability

Not applicable.
